# Supplementary material for: Bee Venom Induces Acute Inflammation through a H2O2-Mediated System That Utilizes Superoxide Dismutase
Source: Toxins (Basel). 2022 Aug 17;14(8):558. doi: 10.3390/toxins14080558 (PMC9414663; doi:10.3390/toxins14080558)
Supplement: Supplementary file 1 [file toxins-14-00558-s001.zip › toxins-1837755-supplementary.pdf]

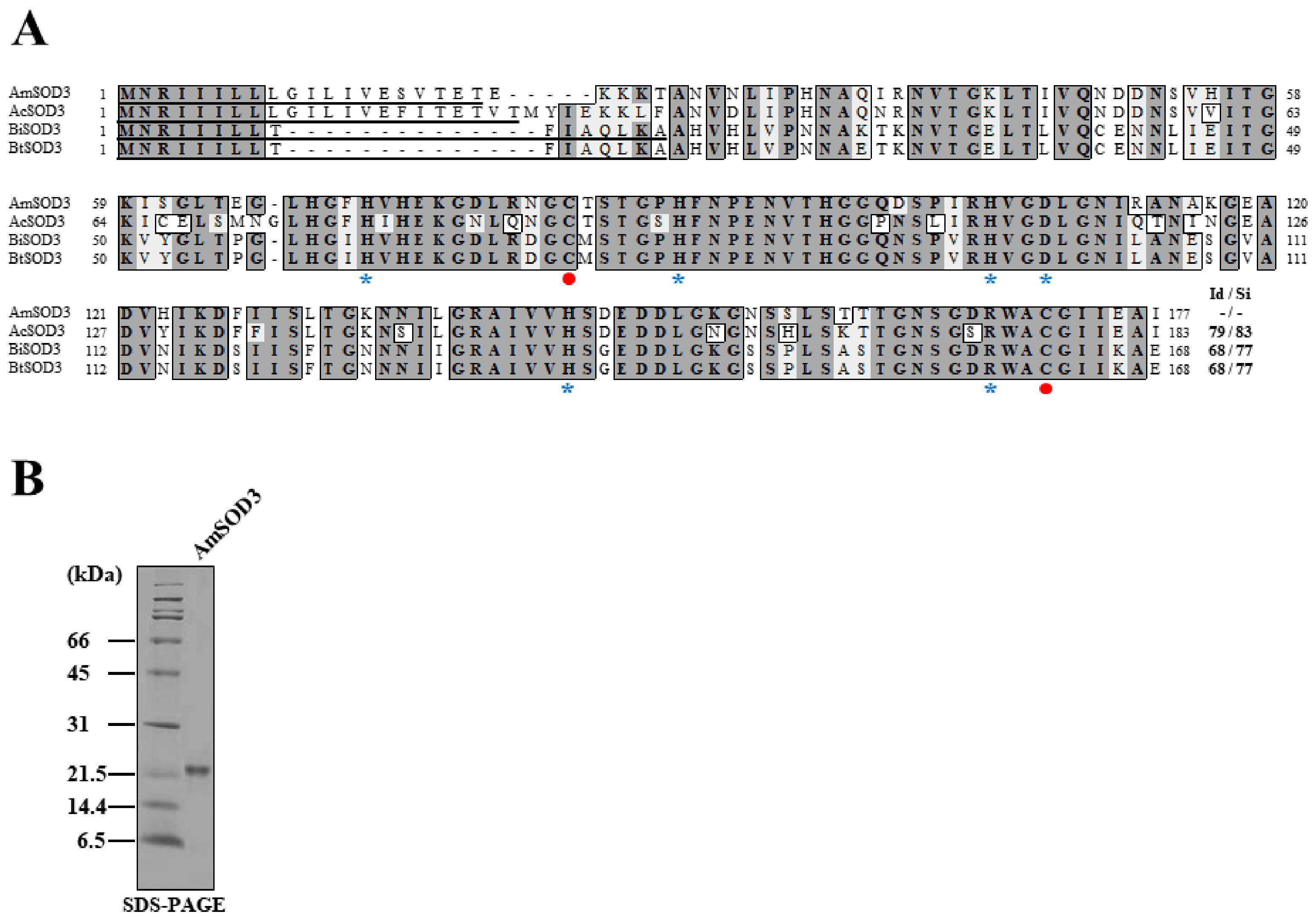

Figure S1. Bee venom SOD3 protein sequences and recombinant AmSOD3 expression. (**A**) Amino acid sequence alignment of bee venom SOD3s. The cleavage site for the predicted signal sequence is underlined. The residues that form disulfide bridges (S-S) and ligate metals (Cu or Zn) are indicated by solid circles and asterisks, respectively. The AmSOD3 sequence was used as a reference for the identity/similarity (Id/Si) values. (**B**) Production of a recombinant AmSOD3 in baculovirus-infected insect cells.

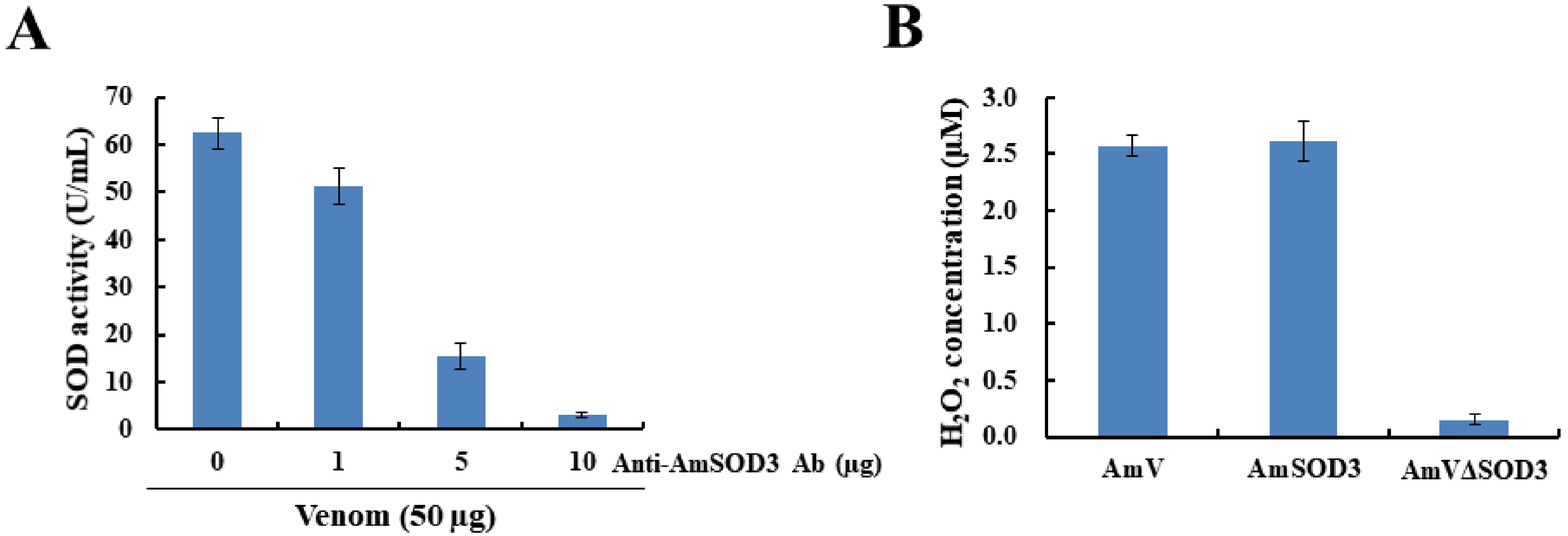

Figure S2. bvSOD3 functions in H_2O_2 formation. (**A**) Preparation of bee venom in which SOD3 was blocked by immunoprecipitation with anti-AmSOD3 antibodies. *A. mellifera* bee venom (50 μg) was incubated with anti-AmSOD3 antibodies (1, 5, and 10 μg) at 37°C for 1 h, and the SOD activity was then measured. The data represent the mean ± SD (*n* = 3). (**B**) In vitro H_2O_2 production by native *A. mellifera* bee venom (AmV), bee venom with blocked SOD3 (AmVΔSOD3), or recombinant AmSOD3 (200 ng). The data represent the mean ± SD (*n* = 3).

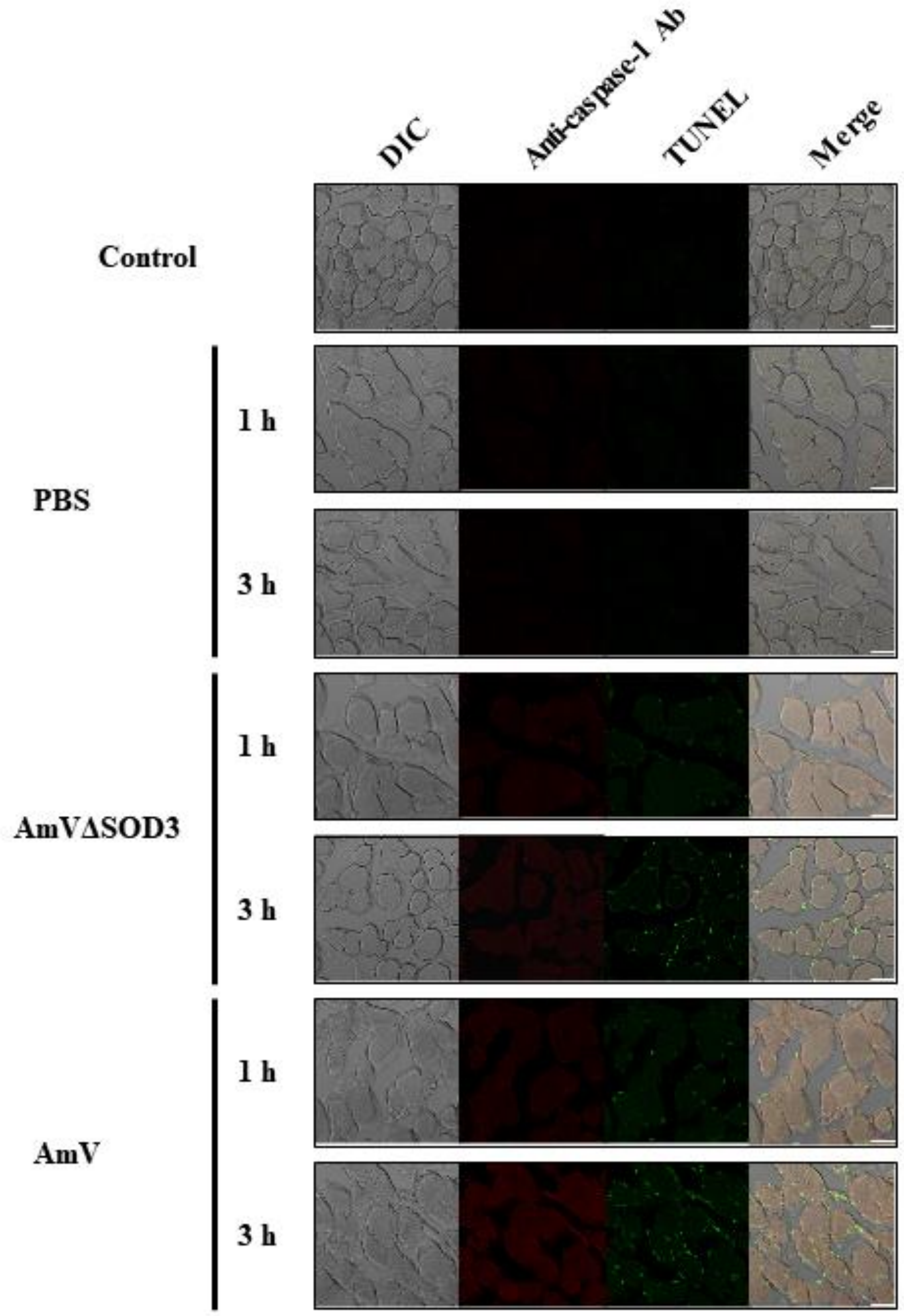

Figure S3. bvSOD3 increases the levels of caspase-1 and cell death. Representative immunofluorescence images showing caspase-1 expression (red) and bee venom-induced cell death (green) in mouse muscles from (Fig. 2A) at 1 or 3 h post injection. Scale bar, 40 μm.

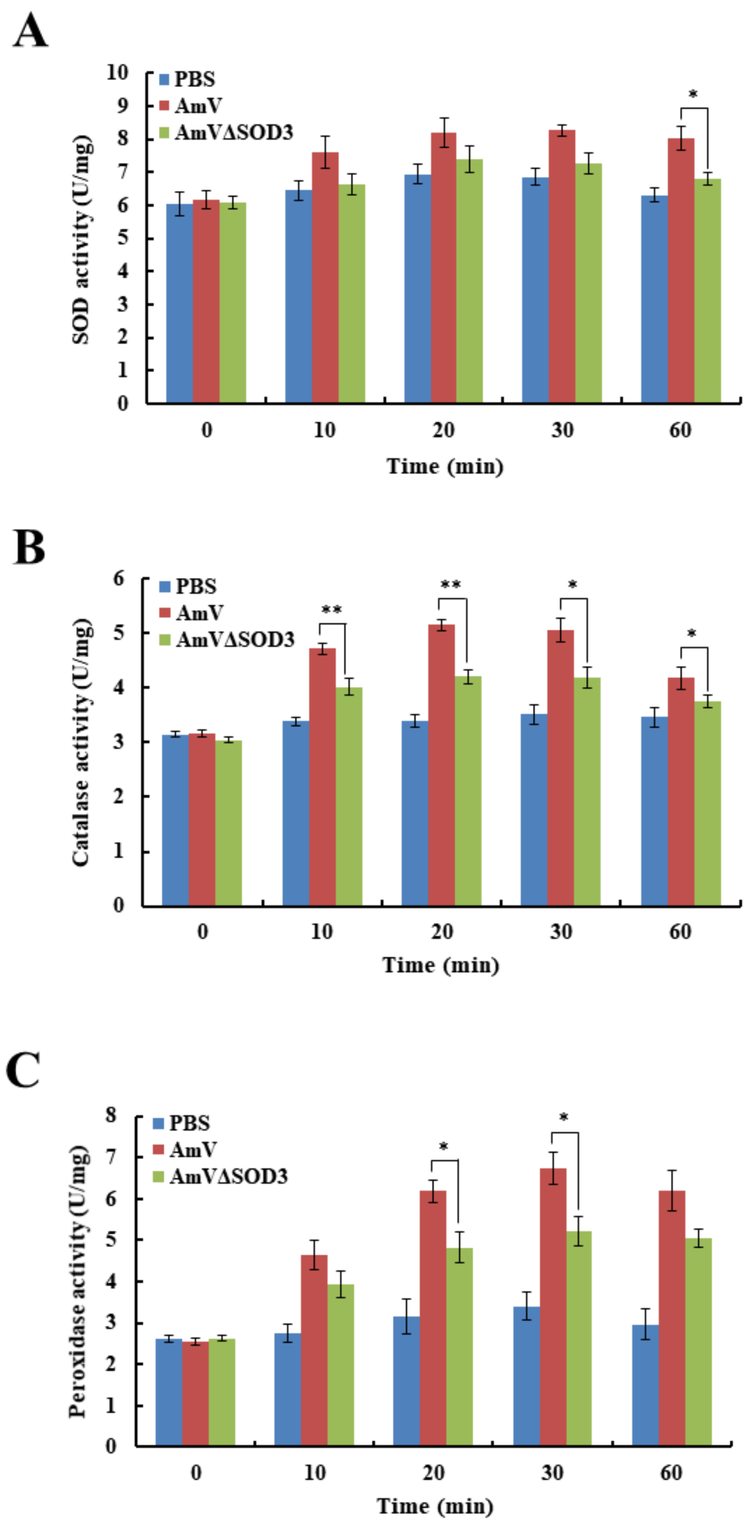

Figure S4: Antioxidant enzyme activity in bee venom-injected mouse muscles. SOD (A), catalase (B), and peroxidase (C) activities in mouse muscle samples analyzed in (Fig. 2A) (n = 5). Data are the mean ± SD; experiments were independently replicated three times. Independent *t*-test, *$P < 0.05$, **$P < 0.01$.

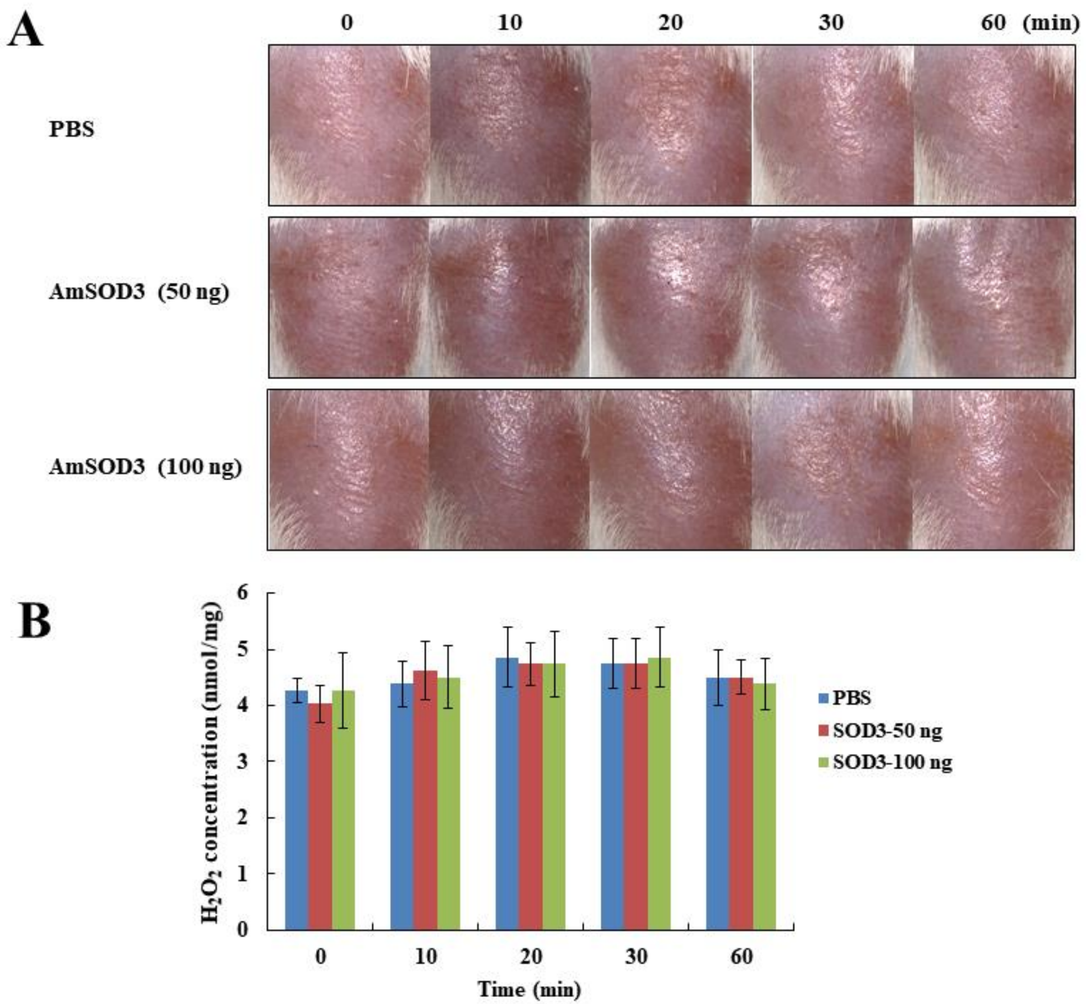

Figure S5. Mice injected with recombinant AmSOD3 alone do not exhibit an inflammatory response or H_2O_2 production. (**A**) *In vivo* effects of recombinant AmSOD3 administration, determined by imaging of representative mice at 10, 20, 30, and 60 min post injection (n = 5). PBS, injection control. (**B**) H_2O_2 concentration in mouse muscles from (A). Data are the mean ± SD; experiments were independently replicated three times.

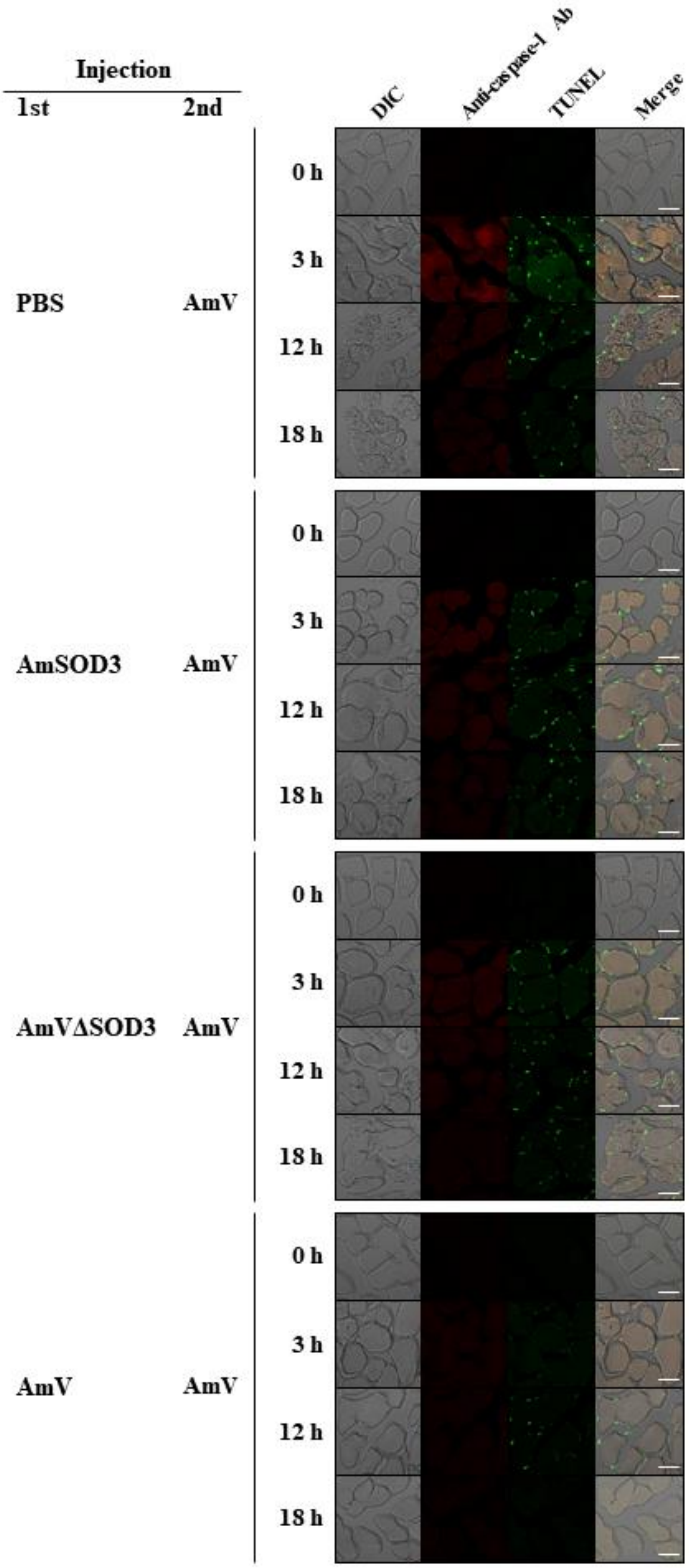

Figure S6. Immunization with bvSOD3 reduces the levels of caspase-1 and cell death. Representative immunofluorescence images showing caspase-1 expression (red) and bee venom-induced cell death (green) in mouse muscles from Figure 4C at 3, 12, or 18 h post challenge. Scale bar, 40 μm.
